# Physiologically based pharmacokinetic modeling and dose adjustment of imipenem in pediatric patients with renal impairment

**DOI:** 10.3389/fcimb.2026.1798911

**Published:** 2026-05-08

**Authors:** Chen Feng, Peng Xiao, Yuchen Qu, Kai Fan, Yueyuan Wang, Xinyun Zhang, Xiaolan Wang, Jie Pan, Yang Deng, Yunli Yu

**Affiliations:** 1 Department of Pharmacy, The Second Affiliated Hospital of Soochow University, Suzhou, Jiangsu, China; 2 College of Pharmacy, Soochow University, Suzhou, Jiangsu, China; 3 Hunan Provincial Key Laboratory of Anti-Resistance Microbial Drugs, The Third Hospital of Changsha, Changsha, Hunan, China

**Keywords:** imipenem, pediatric patients, physiologically based pharmacokinetic model, probability of target attainment, renal impairment

## Abstract

**Objectives:**

To establish a physiologically based pharmacokinetic (PBPK) model of imipenem, predict its exposure in pediatric patients with different renal function, and optimize the dosing regimen.

**Methods:**

GastroPlus™ was used to construct PBPK models for healthy adults, adults with renal impairment (RI), and children with normal renal function, validated by fold error (<2) between predicted and observed pharmacokinetic parameters. Based on the established PBPK models, the exposure of imipenem in pediatric patients with different renal function was predicted. Monte carlo simulations were used to evaluate the probability of target attainment (PTA) for optimized doses and to determine appropriate dosing regimens for pediatric patients with RI.

**Results:**

The PBPK model could adequately predict the exposure of imipenem in different populations after single and multiple administrations (fold error <2). For 15 mg/kg doses, the AUC of imipenem in children with mild RI, moderate RI and severe RI was 1.05-fold, 1.26-fold, and 2.14-fold that of healthy children, respectively. Prolonging infusion from 30 min to 3 h significantly increased PTA. In addition, for susceptible bacteria with the minimum inhibitory concentration (MIC) < 4 mg/L, the recommended doses for pediatric patients aged ≥ 3 years with normal renal function, mild RI, moderate RI, and severe RI were 15, 15, 12, and 7 mg/kg every 6 hours, respectively, with a 3-hour infusion.

**Conclusion:**

This PBPK model can accurately predict the exposure of imipenem in pediatric patients with renal impairment, and the optimized dosing regimen can meet the pharmacodynamic targets, providing support for the precise use of imipenem.

## Introduction

Imipenem is an important member of carbapenem antibiotics, renowned for its broad antimicrobial coverage against gram-positive bacteria, gram-negative bacteria, and anaerobes. Therefore, it is widely used in the treatment of lower respiratory tract infections, abdominal infections, urinary tract infections, sepsis, infective endocarditis, reproductive system infections, bone and joint infections, as well as mixed infections and severe infections caused by unknown pathogens (Zhanel et al., 2007; Papp-Wallace et al., 2011). Additionally, imipenem falls into the category of time-dependent antibiotics. For this agent, the pharmacokinetic/pharmacodynamic (PK/PD) index that shows the strongest correlation with clinical effectiveness is the duration during which the free plasma drug concentration meets or exceeds the minimum inhibitory concentration (MIC). This duration is quantified as a percentage of the dosing interval, denoted as %*f*T > MIC, with a target of at least 40% of the dosing interval (Craig, 1995; Dinh et al., 2022). Multiple clinical studies have shown that for critically ill patients, a higher PK/PD target of 100% *f*T > MIC is required (Bai et al., 2024).

As a hydrophilic drug, imipenem exhibits poor absorption from the gastrointestinal tract, which is why it is primarily delivered via intravenous injection. Imipenem features a short plasma half-life, roughly 1 hour, as well as a low plasma protein binding rate, approximately 20%. It exhibits extensive distribution across a broad range of tissues and body fluids, is capable of crossing the placental barrier, and yet maintains a relatively low concentration in the cerebrospinal fluid (Buckley et al., 1992). It is particularly important to note that when imipenem is administered alone, it is rapidly metabolized into an inactive metabolite by enzyme dehydropeptidase (DHP-1) in the brush border of the kidney, thereby losing its therapeutic effect (Mouton et al., 2000; Wang et al., 2024). In view of this, imipenem is usually administered as a combined preparation with cilastatin, a DHP-1 inhibitor, in a 1:1 ratio. After administration of the imipenem-cilastatin combination, approximately 70% of the unchanged imipenem is excreted through the kidneys, which can effectively enhance the therapeutic effect and reduce renal toxicity (Buckley et al., 1992).

As is well known, imipenem is mainly excreted from the body via the kidneys (Barza, 1985; Buckley et al., 1992). Therefore, changes in renal function can affect the PK of imipenem. According to the label of imipenem, the recommended doses of imipenem are 0.5 g every 6 hours, 1 g every 8 hours, or 1 g every 6 hours. When the creatinine clearance is less than 90 mL/min, the dose of imipenem must be reduced. Thus, the label of imipenem provides detailed recommendations for patients with renal insufficiency based on creatinine clearance (FDA, 2018). With the widespread use of imipenem in adult patients, it has also gradually been widely used in pediatric patients as well (Wong et al., 1991; Asensi et al., 1993; Lockowitz et al., 2025). According to the label, for children over 3 months of age with normal renal function, the recommended dosage of imipenem is 15–25 mg/kg every 6 hours (FDA, 2018). However, there are no clear recommended dosages in the label for children with impaired renal function, which is understandable. Prospective PK studies characterizing drug disposition during children’s development are hindered by multiple factors, including more complex trial design, ethical approval challenges, and barriers to informed consent (Kern, 2009; Laughon et al., 2011; Zimmerman et al., 2015). The PK/PD characteristics of imipenem in children with altered renal function have not been fully elucidated. Therefore, the label of imipenem lacks sufficient evidence to provide dosage recommendations for children with impaired renal function. However, the widespread use of imipenem in the pediatric population makes it necessary to further study its PK/PD characteristics in children with altered renal function, which will help optimize the dosage regimen in this population.

Physiologically based pharmacokinetic (PBPK) models can identify the impact of various physiological and biochemical characteristics on drug disposition, and may serve as a useful tool for predicting the PK of pediatric patients with renal impairment (RI) (Huang and Rowland, 2012; Emoto et al., 2020). In recent years, PBPK models have become a valuable platform for predicting PK exposure in special populations (Templeton et al., 2018; Whang et al., 2022; Wu et al., 2025). By integrating information related to disposition in different human organ systems and drug-specific clinical pharmacology information, PBPK models simulate drug exposure data in virtual populations (Sager et al., 2015). These simulated data can provide references for dosage regimen recommendations and dose optimization in special populations. To date, although several population PK models of imipenem have been reported (Dailly et al., 2003; Dinh et al., 2022; Bai et al., 2024; Wang et al., 2024), a PBPK model of imipenem has not yet been established in pediatric patients with varying renal function. The aim of this study is to establish and validate a PBPK model for imipenem, use this model to further investigate and predict imipenem exposure in pediatric patients with different levels of renal function, and develop a simulation-based dosing regimen. We hope this can provide a theoretical basis for the individualized administration of imipenem in pediatric patients with RI.

## Methods

### Software

GastroPlus™ software (version 9.9; SimulationsPlus, Inc., Lancaster, CA) was used to construct the PBPK model in this study. Population estimates from the age-related physiology module were utilized to generate human physiological parameters, and the model input parameters were optimized in GastroPlus™. GetData Graph Digitizer 2.26 (S. Fedorov) was employed to digitize the original observed clinical values.

### Model development and evaluation

**Figure 1:**
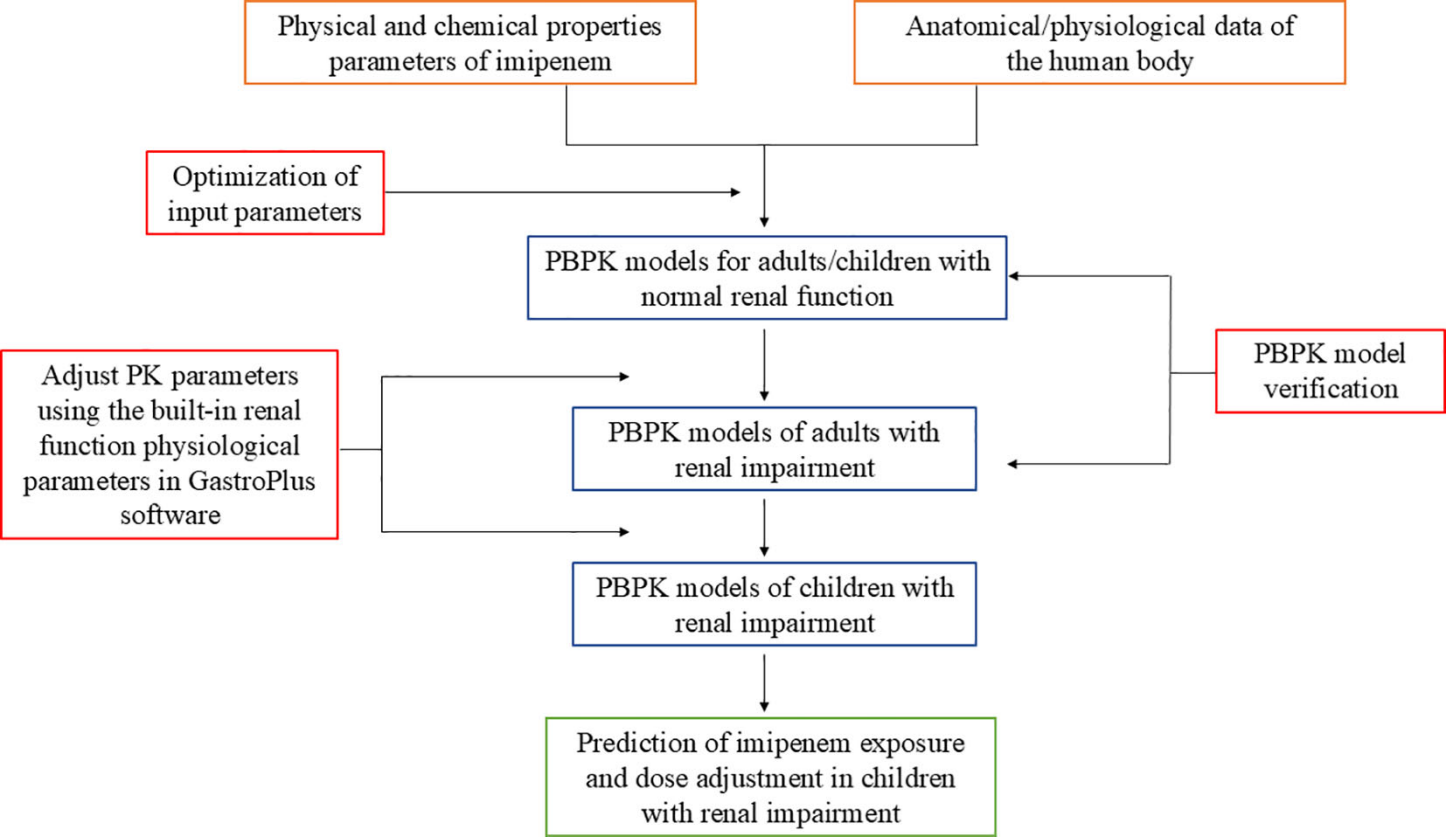
The development process of imipenem PBPK model.

The development process and application of the imipenem PBPK model are shown in **Figure 1**. The physicochemical parameters of imipenem used for modeling are listed in **Table 1**, including molecular weight, Log P, pKa, solubility and plasma unbound fraction (Fup), all of which were obtained from the literature or the DrugBank database. The sources of clinical trial data are detailed in **Supplementary Table 1**. All included clinical studies utilized either imipenem/cilastatin or a combination formulation of imipenem with an enzyme inhibitor, and all reported the plasma concentration data of imipenem co-administered with the enzyme inhibitor. We digitally extracted the imipenem concentration data for PBPK modeling and validation using GetData Graph Digitizer 2.26 software.

**Table 1:** Physicochemical parameters of imipenem used in the PBPK model.

Parameter	Initial value	Source
Molecular formula	C_12_H_17_N_3_O_4_S	DrugBank
MW (g/mol)	299.35	DIDB
LogP	−3.9	DrugBank
pKa (Acid)	3.44	DrugBank
pKa (Base)	10.88	DrugBank
Solubility (mg/mL)	0.78	DrugBank
Fup (%)	80	DIDB
CLr (L/h)	10.71	DIDB

MW, molecular weight; LogP, apparent partition ratio; pKa, acid dissociation constant; Fup, fraction unbound in plasma. CLr, renal clearance; DIDB, University of Washington drug interaction database, https://www.druginteractionsolutions.org/solutions/drug-interaction-database; Drug Bank, drug bank database, https://www.drugbank.ca/. *The renal clearance of imipenem is the clearance when used in combination with cilastatin.

Multiple methods were employed to evaluate the performance of the imipenem PBPK model. First, the predicted concentration-time curves were compared with the observed data from the respective clinical studies. In addition, goodness-of-fit (GOF) plots were generated to compare the deviations between predicted concentration data and the corresponding clinically observed values. More importantly, the PBPK model was further evaluated by analyzing the errors between the observed and predicted PK parameters, with a specific focus on the area under the concentration-time curve (AUC) and the maximum plasma concentration (C _max_). Fold error (predicted value/observed value), a widely accepted metric in the pharmaceutical industry, was used to assess prediction accuracy. Predictions were considered acceptable when the fold error was less than 2.

### Adult PBPK model development

A PBPK model for healthy adults was established based on clinical trial data, and simulation information was manually set, including subjects’ demographic information (age, weight, gender), administration dose (weight-dependent dose and fixed dose), administration route (intravenous infusion), and administration time (0.5 h or 2.0 h). Both single-dose and multiple-dose intravenous infusions of imipenem were simulated. The performance of the model was evaluated using the corresponding clinical PK values.

### Model for adult patients with RI

The physiological parameters associated with RI, including glomerular filtration rate (GFR), plasma protein concentration, blood pressure, and hematocrit, have been integrated into GastroPlus™ software. We employed the software’s built-in algorithm Fup*GFR to estimate the renal clearance (CL_R_) of imipenem. According to the guidelines of FDA, renal function classification is determined by GFR values, where GFR ≥90, 60-89, 30-59, and 15–29 mL/min/1.73 m² correspond to normal renal function, mild RI, moderate RI, and severe RI, respectively. We established PBPK models for adults with three degrees of renal insufficiency. Subsequently, the predictive accuracy of these PBPK models was validated through a single intravenous infusion of 250 mg (**Supplementary Table 1**).

### Model for pediatric patients with normal renal function

The study utilized an adult physiologically based PBPK model as the foundation. Within the GastroPlus™ software, physiological parameters and anthropometric data for pediatric populations, including organ weights, blood flow rates, cardiac output, *in vivo* CL_R_, and plasma protein concentrations, were generated using weight- and age-dependent algorithms derived from the software’s default database. These data were employed to construct the pediatric PBPK model, while other input parameters (e.g., physicochemical properties) remained consistent with the adult model. We assumed that the renal excretion mechanism of imipenem is identical in adults and children, with GFR calculated using the default algorithm in GastroPlus™. Simulations were conducted for single-dose (15 mg/kg, 25 mg/kg) intravenous administration regimens in pediatric populations. The established pediatric PBPK model was validated using clinical data from references (Claesson et al., 1992; Bradley et al., 2023) (**Supplementary Table 2**).

### Population simulation

To investigate the effects of individual variability on population-level physiological characteristics and compound-related variables, a population simulator module (with a simulated sample size of 200, including a 1:1 male-to-female ratio) was utilized to perform population PK simulations of imipenem. For each clinical trial, demographic data and dosing regimens consistent with those reported in the literature were integrated into the software. The dosing regimen employed in the population simulations was aligned with that of the original clinical study, and the 90% confidence interval (90% CI) of the concentration-time curve was analyzed.

### PBPK model sensitivity analysis

To identify the most sensitive parameters in the model, a global sensitivity analysis was performed. By simulating the PK profiles of average individuals in healthy conditions, mild RI, moderate RI, and severe RI following a 30-minute intravenous infusion of 500 mg imipenem, we analyzed the sensitivity of the model parameters to the predicted area under the concentration-time curve from time zero to infinity (AUC _0-inf_) and maximum plasma concentration (C _max_) of imipenem. All evaluated parameters were varied by ±10%, and the relative changes in AUC_0-inf_ and C _max_ were expressed as percentages. For example, a relative change of -5% indicates that a 10% increase in the parameter results in a 5% decrease in AUC_0-inf_ or C _max_.

### Prediction of imipenem exposures in pediatric patients with RI

According to the WHO classification for children, children aged 3–6 years are defined as preschool children, those aged 6–12 years as school-age children, and those aged 12–18 years as adolescents. After validating the PBPK model of imipenem in pediatric patients with normal renal function, we used this model to predict the PK characteristics of imipenem following intravenous administration at a dose of 15 mg/kg in three representative pediatric patients with renal impairment (3 years, 8 years, and 16 years old). The software-built-in parameters were used to simulate pediatric patients with different degrees of RI, subsequently, other physiological parameters were automatically adjusted based on renal function. Meanwhile, the predicted values of key PK parameters were compared against their corresponding counterparts in pediatric patients with normal renal function, who were designated as the control group. For pediatric patients with RI, the recommended dose was derived by multiplying the geometric mean ratio of PK parameters between pediatric populations with RI and healthy pediatric populations by the standard dose administered to healthy pediatric populations.

### Pharmacodynamic evaluation of imipenem

Similar to meropenem, imipenem exhibits time-dependent bactericidal activity against susceptible bacteria. The PD exposure—measured as the percentage of time that free drug concentration exceeds the minimum inhibitory concentration (%*f*T > MIC)—is the PK/PD parameter associated with therapeutic efficacy. For carbapenems, the PK/PD target of “50%-100%*f*T > MIC” has been well validated (Abdul-Aziz et al., 2020). In previous pharmacokinetic studies, “70%*f*T > MIC” has also been used as an efficacy indicator to achieve maximum bactericidal activity (Kuti et al., 2004). Given the need for a rigorous endpoint to ensure reliable efficacy, this study set the target for probability of attaining the bactericidal pharmacodynamic target at “70%*f*T > MIC”, which represents a more conservative threshold. To determine a suitable dosing regimen for imipenem in pediatric patients with varying renal function, this study employed Monte Carlo simulation to calculate the probability of target attainment (PTA) for each imipenem dosing regimen; a dosing regimen was deemed acceptable if its PTA was ≥ 80%. Additionally, we also evaluated the effects of 1-hour and 3-hour intravenous infusions of imipenem.

## Results

### Imipenem PBPK model for healthy population

The simulated and observed mean plasma concentration-time profiles of imipenem following 7 single-dose administrations and 2 multiple-dose administrations are shown in **Supplementary Figure 1**, respectively. In children with normal renal function, the simulated and observed plasma concentration-time profiles following a single intravenous infusion of 15 mg/kg and 25 mg/kg are shown in **Supplementary Figure 2**. The theoretical CL_R_ of imipenem in healthy adults and children with normal renal function was calculated as the product of the Fup and GFR. **Supplementary Table 3** shows that the individual clearance rates of adults simulated by GastroPlus™ range from 10.726 to 11.176 L/h, and those of children range from 7.886 to 8.489 L/h. The estimated apparent volume of distribution at steady state was about 3.259 L/kg in children, compared with 11.663 L/kg in adults. As the administered dose increased, the predicted and observed PK parameters (AUC_0-t_, C_max_) also increased accordingly. **Tables 2**, **3** show that for intravenous infusion of imipenem at the same dose, prolonging the infusion duration could reduce the peak concentration but had a minimal effect on predicted AUC_0-t_. In addition, the fold-error results demonstrated that the predicted and observed PK parameters were in good agreement in normal adults and children, with all values falling within the 2-fold error range (0.79–1.27 fold). The GOF plot (**Supplementary Figure 4**) showed that more than 90% of the individual observed plasma concentrations were within the 2-fold error ranges of the predicted values, demonstrating the robustness of the PBPK model.

**Table 2:** Comparison between observed and predicted pharmacokinetic parameters of imipenem with different dose regimens in adults.

Renal function	Dose	Infusion time (h)	AUC_0-t_ (μg·h/mL)			C_max_ (μg/mL)		
			Observed	Predicted	Fold-Error	Observed	Predicted	Fold-Error
Normal	0.25 g (iv drip)	0.5	25.48	22.59	0.87	20.05	19.21	0.95
Normal	0.5 g (iv drip)	0.5	60.65	50.91	0.84	48.38	43.62	0.90
Normal	1.0 g (iv drip)	0.5	89.37	88.47	0.99	65.64	71.15	1.08
Normal	0.5 g (iv drip)	2.0	57.20	46.20	0.81	21.23	20.56	0.97
Normal	1.0 g (iv drip)	2.0	116.81	92.39	0.79	42.33	41.12	0.97
Normal	0.5 g (iv)	–	42.35	43.86	1.04	–	165.99	–
Normal	1.0 g (iv)	–	95.75	87.72	0.92	–	331.99	–
Normal	0.25g (iv drip)	0.15	–	22.33	–	–	23.09	–
Mild RI	0.25g (iv drip)	0.15	19.97	23.13	1.16	–	23.03	–
Moderate RI	0.25g (iv drip)	0.15	29.80	37.69	1.26		25.67	–
Severe RI	0.25g (iv drip)	0.15	60.76	66.65	1.10		25.87	–

AUC_0-t_, area under the plasma concentration-time curve from time zero extrapolated to simulation end time; Cmax, maximum concentration of drug in blood plasma; RI, renal impairment.

**Table 3:** Comparison between observed and predicted pharmacokinetic parameters of imipenem with different dose regimens in pediatric patients.

Renal function	Dose (Delivery Path)	Infusion time (h)	AUC_0-t_ (μg·h/mL)			C_max_ (μg/mL)		
			Observed	Predicted	Fold-Error	Observed	Predicted	Fold-Error
Normal	15 mg/kg (iv drip)	0.5 h	29.70	37.83	1.27	–	52.72	–
Normal	25 mg/kg (iv drip)	0.5 h	58.14	63.04	1.08	–	87.87	–
Normal	15 mg/kg (iv drip)	1.0 h	56.17	47.40	0.84	35.38	40.10	1.13
Normal*	15 mg/kg (iv drip)	0.5 h	–	42.82	–	–	56.42	–
Mild RI*	15 mg/kg (iv drip)	0.5 h	–	44.73	–	–	57.37	–
Moderate RI*	15 mg/kg (iv drip)	0.5 h	–	53.77	–	–	61.14	–
Severe RI*	15 mg/kg (iv drip)	0.5 h	–	91.44	–	–	71.58	–

AUC_0-t_, area under the plasma concentration-time curve from time zero extrapolated to simulation end time; Cmax, maximum concentration of drug in blood plasma; RI, renal impairment. *Represents the PK parameters of an 8−year−old school−age child after administration of imipenem.

### Imipenem PBPK model for adult RI patients

Incorporating the changes in key physiological parameters associated with RI into the model, the mean blood concentration–time curve following a single 250 mg intravenous infusion of imipenem was predicted and compared with the observed data from clinical studies (Gibson et al., 1985)(**Supplementary Figure 3**). In the corresponding clinical trials, the predicted PK parameters (AUC_0-t_, C_max_) showed high agreement with the observed data, with all errors falling within 1.26-fold of the observed values (**Table 2**). In adult patients with RI, the elimination of imipenem was significantly delayed, leading to a marked increase in the AUC_0-t_. Specifically, compared with individuals with normal renal function, patients with mild, moderate, and severe RI exhibited 1.03-fold, 1.69-fold, and 2.98-fold higher AUC values, respectively. In contrast, no significant intergroup differences were observed in the C _max_ (**Table 2**). In conclusion, the observed results demonstrate that the refined PBPK model accurately simulates imipenem exposure in adult patients with renal impairment and can be further extrapolated for imipenem pharmacokinetic simulations in pediatric patients with renal impairment.

### Population simulation for adults and children

**Figure 2:**
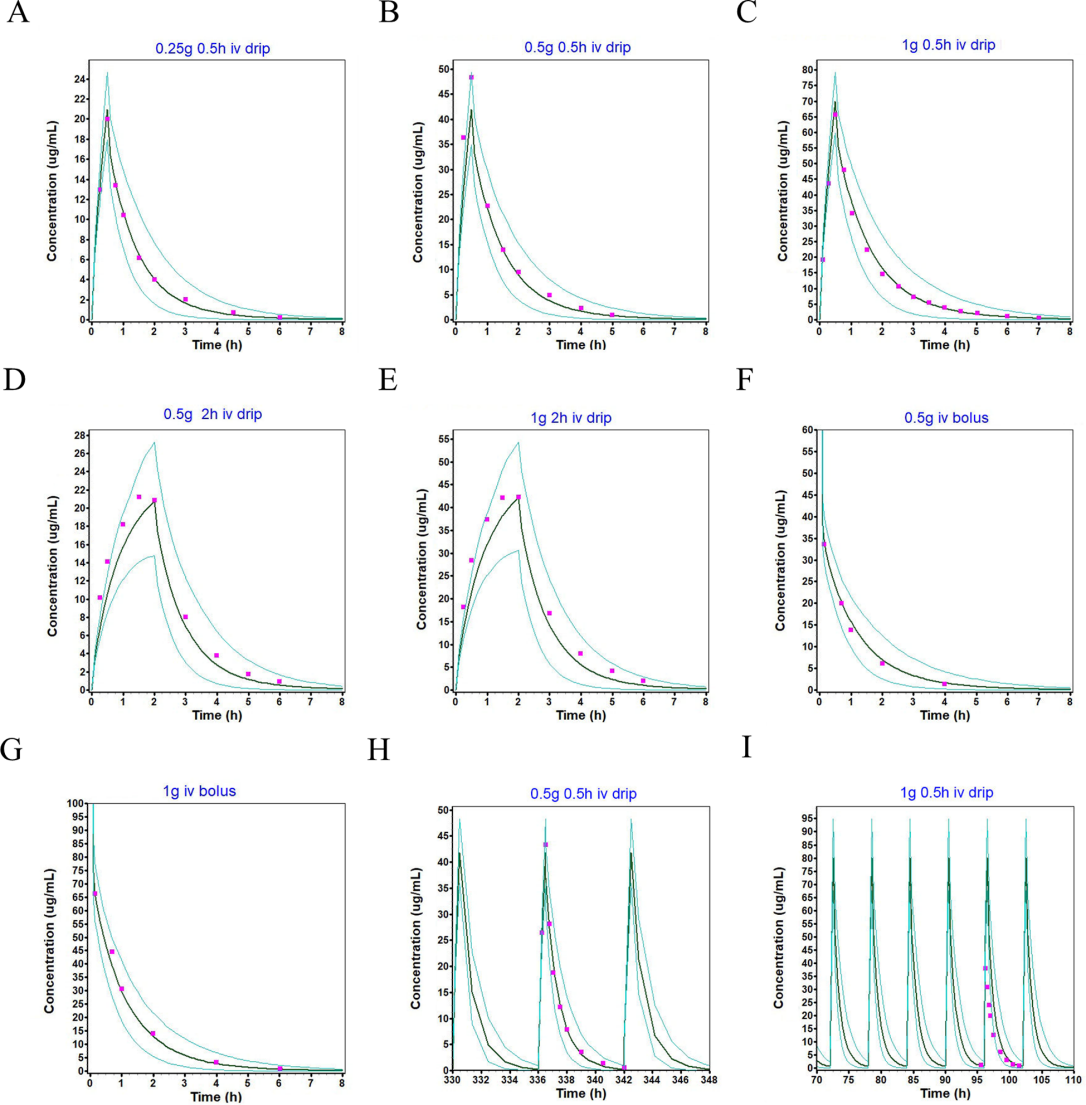
Population simulations of imipenem following intravenous infusion in healthy adults are presented in **(A-I)**. In these simulations, the dark green line represents the population simulation results, the light green line denotes the plasma concentration-time profiles with a 90% probability interval, and the purple squares indicate the observed values.

**Figure 3:**
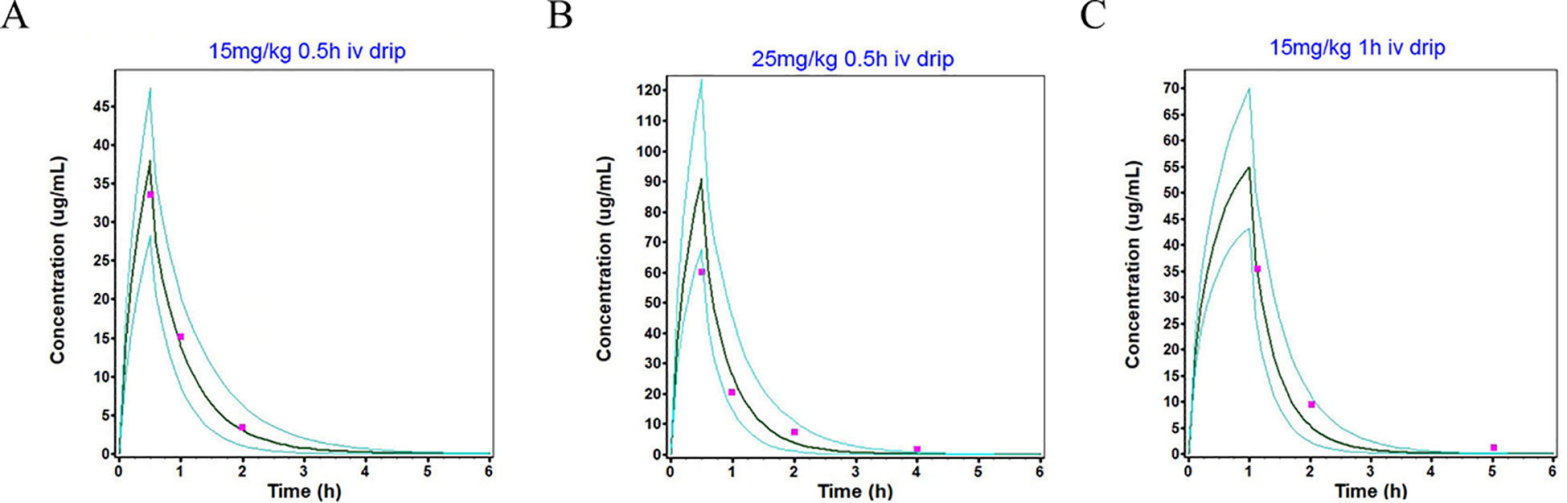
Population simulations of imipenem following intravenous infusion in healthy children are presented in **(A-C)**. In these simulations, the dark green line represents the population simulation results, the light green line denotes the plasma concentration-time profiles within a 90% probability interval, and the purple squares indicate the observed values.

**Figure 4:**
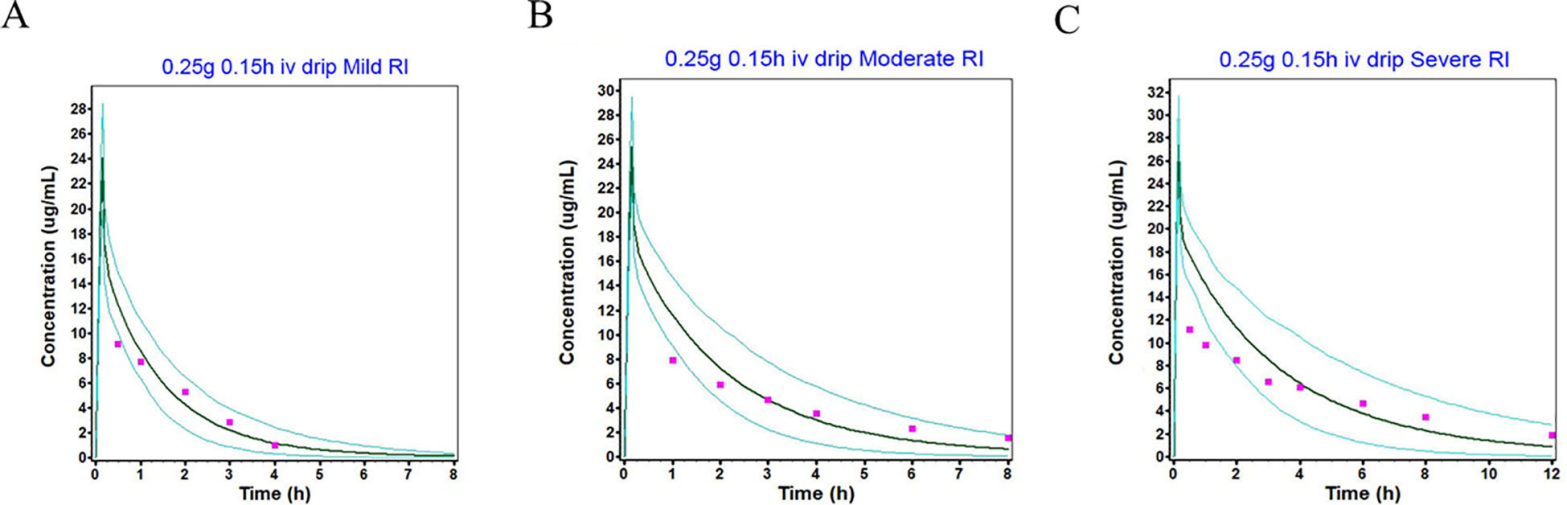
Population simulations of imipenem following intravenous infusion in adults with mild RI **(A)**, moderate RI **(B)**, and severe RI **(C)** are presented. In these panels, the dark green line represents the population simulation results, the light green line denotes the plasma concentration-time profiles within a 90% probability interval, and the purple squares indicate the observed values.

The population simulation results of different single-dose and multiple-dose regimens for intravenous administration of imipenem in healthy adults and children are shown in the **Figures 2**, **3**, presenting the simulated mean plasma concentration-time curves and observed clinical values. **Figure 4** shows the population simulation results in adults with renal impairment. All figures exhibited the clinically observed values alongside the simulated mean plasma concentration-time profiles, with nearly all simulated and observed data points falling within the 90% confidence interval. Specifically, all observation data are within the minimum and maximum data of the corresponding population simulation. These results indicated that the observed values could be adequately represented by the drug concentration-time curves of the simulated population.

### Imipenem PBPK model sensitivity analysis

To identify the most sensitive parameters in the model, a sensitivity analysis was performed based on the simulation of an intravenous infusion of imipenem 500 mg over 30 minutes. The parameters included in the analysis were: plasma unbound fraction (Fup), renal clearance, tissue-to-plasma partition coefficient (Kp), and ratio of plasma protein binding (Rbp). Model prediction results for healthy population and patients with RI showed that the pharmacokinetic parameter AUC_0-inf_ was relatively sensitive to Fup and renal clearance, while C_max_ was relatively sensitive to Fup and Rbp. Detailed results of the sensitivity analysis are presented in **Supplementary Figure 5**. When Fup was adjusted from 80% to 72% and 88%, the relative changes in both pharmacokinetic parameters (AUC_0-inf_ and C_max_) were less than 10%. A 10% increase in renal clearance resulted in only a slight reduction in AUC_0-inf_ and C_max_ by approximately 8% and 1%, respectively, indicating that renal clearance exerts a more pronounced effect on AUC_0-inf_ than on C_max_. As shown in **Supplementary Figure 5**, with the progression of RI, the sensitivity of AUC_0-inf_ to Kp and Rbp further decreased, and the sensitivity of C_max_ to Fup also decreased.

### Prediction of imipenem exposures in pediatric patients with RI

**Figure 5:**
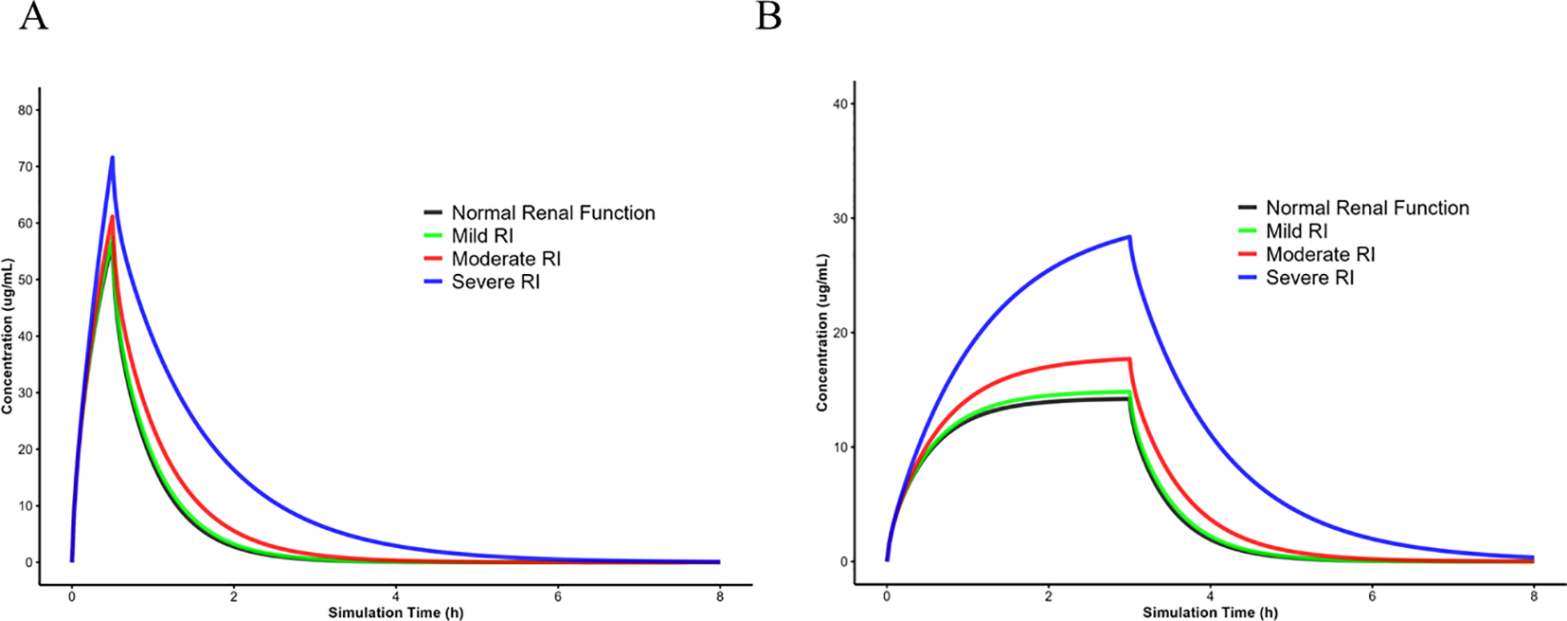
Predicted mean plasma concentration-time profiles of imipenem in school-age children with differing renal function following intravenous administration of 15 mg/kg dose via 30-minute **(A)** and 3-hour infusions **(B)**.

Based on the recommended dosage of imipenem for children with normal renal function, this study predicted imipenem exposure in preschool children, school-age children, and adolescents with RI following intravenous administration of 15 mg/kg. The concentration-time curve of imipenem in a representative school-age child is presented in **Figure 5**. As shown in the predicted results in **Table 4**, compared with preschool children with normal renal function, the AUC_0-t_ values in preschool children with mild, moderate, and severe RI were 1.03-fold, 1.19-fold and 2.01-fold higher, respectively. Similarly, compared with school-age children with normal renal function, the AUC_0-t_ values in school-age children with mild, moderate, and severe RI were 1.05-fold, 1.25-fold and 2.14-fold higher, respectively. In adolescents, the AUC_0-t_ values in those with mild, moderate, and severe RI were 1.05-fold, 1.34-fold and 2.36-fold higher than those in adolescents with normal renal function, respectively.

**Table 4:** Recommended dose adjustments for imipenem in RI pediatrics.

Parameter	Normal children	Mild RI	Moderate RI	Severe RI
Preschool Children				
AUC_0-24h_ (μg h/mL)	31.358	32.581	38.928	63.158
Geometric mean ratio (RI/healthy)		1.03	1.19	2.01
School-age Children				
AUC_0-24h_ (μg h/mL)	42.818	44.731	53.765	91.441
Geometric mean ratio (RI/healthy)		1.05	1.25	2.14
Adolescents				
AUC_0-24h_ (μg h/mL)	86.804	91.535	116.39	204.56
Geometric mean ratio (RI/healthy)		1.05	1.34	2.36
Proposed recommended dose	15 mg/kg q6h	15 mg/kg q6h	12 mg/kg q6h	7 mg/kg q6h

AUC_0-24h_, area under the plasma concentration-time curve from time zero extrapolated to 24h; RI, renal impairment; q6h, 6-hour intervals.

Analysis of imipenem exposure in pediatric patients with RI revealed that no dosage adjustment was required for children with mild RI, whereas corresponding dosage adjustments were necessary for those with moderate and severe RI. The proposed dosage for pediatric patients with different degrees of RI was estimated by multiplying the standard dosage for healthy children by the geometric mean ratio of pharmacokinetic parameters between pediatric patients with RI and those with normal renal function. Therefore, based on the standard dosage of 15 mg/kg every 6 hours for children with normal renal function, the recommended dosage for children aged ≥3 years with moderate and severe RI was adjusted to 12 mg/kg every 6 hours and 7 mg/kg every 6 hours, respectively (**Table 4**).

### Pharmacodynamic evaluation of imipenem

**Figure 6:**
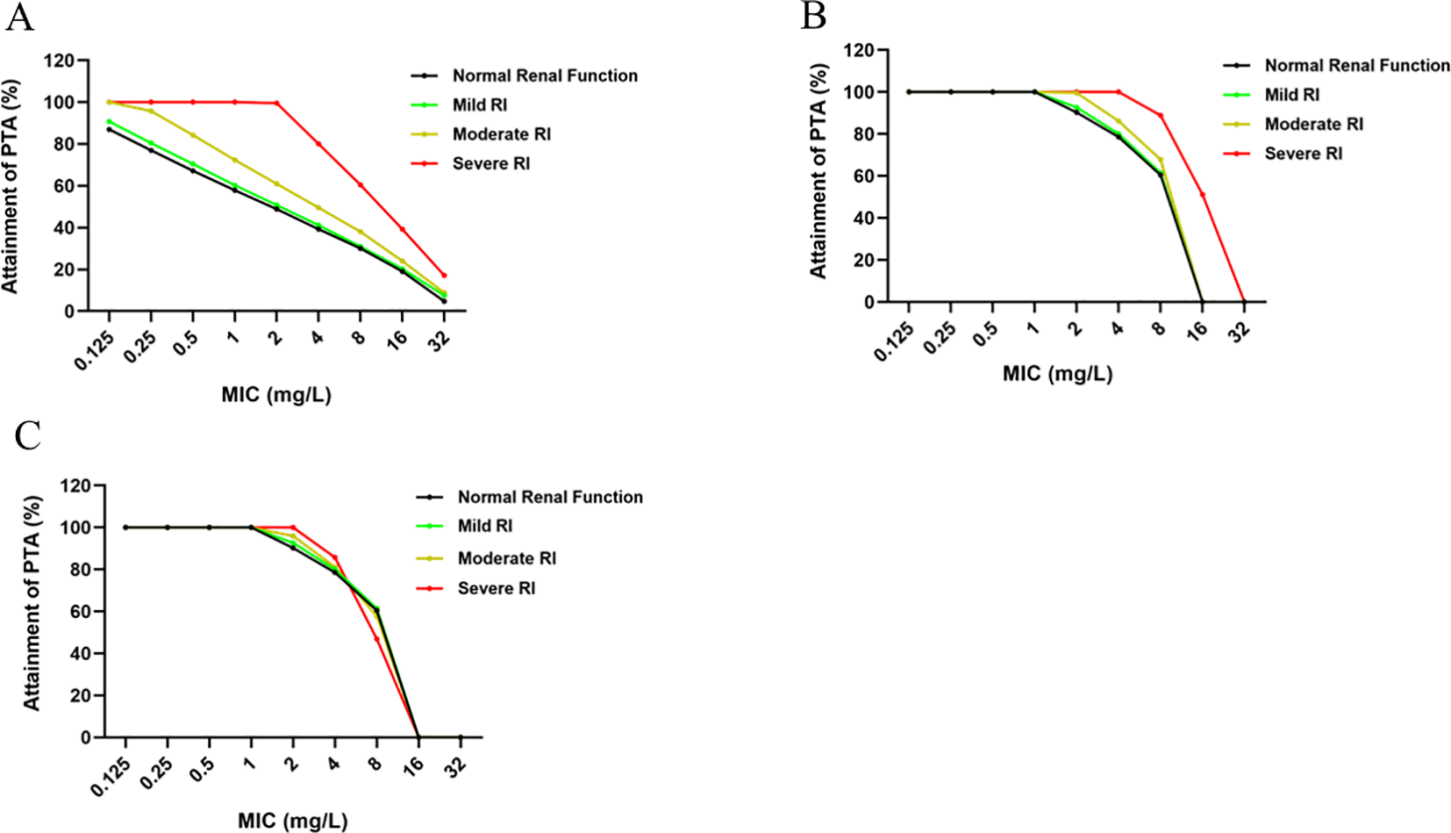
PTA for PK/PD indices in pediatric patients with differing renal function, following administration of a 15 mg/kg dose via intravenous infusion over 30 minutes **(A)** and 3 hours **(B)**, respectively. **(C)** shows the probability of PTA in pediatric patients with normal renal function, mild RI, moderate RI, and severe RI following a 3-hour intravenous infusion of the respective doses of 15 mg/kg, 15 mg/kg, 12 mg/kg, and 7 mg/kg.

When the recommended dose of imipenem was 15 mg/kg with a 0.5-hour infusion, 48.81%, 50.71%, 60.95%, and 99.52% of children with normal renal function, mild RI, moderate RI, and severe RI, respectively, achieved the target of 70%*f*T > MIC at the MIC of 2 mg/L. In contrast, when the MIC was 4 mg/L, the proportions of children meeting this target decreased to 39.28%, 41.19%, 49.52%, and 80%, respectively (**Figure 6A**). Prediction results indicated that for the imipenem dosing regimen of 15 mg/kg every 6 hours with a 0.5-hour infusion, PTA was relatively low in children with normal renal function, mild RI, and moderate RI. After extending the imipenem infusion duration to 3 hours, the predicted plasma concentration-time curve was shown in **Figure 5B**. At a MIC of 4 mg/L, the PTAs were 78.57%, 80%, 86.19%, and 100% for children with normal renal function, mild RI, moderate RI, and severe RI, respectively (**Figure 6B**). This demonstrated that prolonging the infusion time significantly increased the PTA in children with different renal function statuses against pathogens with the MIC of 4 mg/L. Subsequently, based on the study results, the dose of imipenem was adjusted as follows: 15 mg/kg (normal renal function), 15 mg/kg (mild RI), 12 mg/kg (moderate RI), and 7 mg/kg (severe RI), administered every 6 hours with a 3-hour infusion. At the MIC of 4 mg/L, the PTAs in children with different renal function statuses were 78.57%, 80%, 81.19%, and 85.71%, respectively (**Figure 6C**). These findings suggested that even though the doses were reduced in children with moderate and severe RI, the relevant pharmacodynamic targets could still be achieved, indicating that such dose adjustments are acceptable.

## Discussion

Owing to the paucity of pediatric data, uncertainties persist regarding the specific impact of growth and development on PK/PD profiles in pediatric patients, as well as the appropriate drug dosages for this population (Samant et al., 2015). Furthermore, the information provided in drug package inserts regarding pediatric populations and other special populations is often insufficient. Pharmaceutical research based on PBPK models exerts a profound impact on treatment strategies for special populations, enabling accurate assessment of drug distribution, as well as the benefits and risks associated with drug administration regimens (Chen et al., 2020). PBPK models can maximize the utilization of available data and, through data extrapolation, supplement and enhance drug-related information for special populations under different disease states (Yang et al., 2024). In this study, we used GastroPlus™ software to construct and validate the PBPK models of imipenem in healthy adults and children. Concurrently, a PBPK model for imipenem in adults with RI was established. Based on the physiological parameters and drug property parameters of the validated PBPK models, physiological parameters such as age and renal function were adjusted, and the constructed PBPK models were extrapolated to pediatric patients with RI to individualize and optimize the imipenem dosage for these renal-impaired pediatric patients. The models developed in this study have undergone comprehensive evaluation and can adequately describe the *in vivo* PK processes of intravenously administered imipenem in both adult and pediatric patients.

It is well-known that the clearance of imipenem decreases significantly in cases of renal insufficiency. This phenomenon is not unexpected, as imipenem is mainly eliminated via the kidneys (Barza, 1985; Zhanel et al., 2007). It has been reported that the antibacterial effect of antibiotics is mainly closely related to the free drug concentration of antibiotics (Roberts et al., 2013). Therefore, the unbound fraction of imipenem in plasma is incorporated into the present model. According to literature reports, the unbound fraction of imipenem in healthy individuals is approximately 0.8. However, there is currently no specific data on the unbound fraction of imipenem in patients with RI. In GastroPlus™ software, as the degree of renal impairment increases in patients with renal insufficiency, the default unbound fraction shows an upward trend (**Supplementary Table 3**), which may be related to the decrease in serum albumin concentration associated with renal diseases (Keller et al., 1984). In this study, a free fraction of 0.8 was adopted for healthy individuals, while the default value of the software was used for patients with renal insufficiency. Besides, the *in vivo* CL_R_ was calculated using the formula “Fup*GFR” in this study, and non-renal clearance pathways were not considered during the model construction process (Zhou et al., 2021). This algorithm has been applied in dose extrapolation studies for various special populations (Zhou et al., 2021; Dinh et al., 2022). According to the PBPK model established by us, the theoretical renal clearance in healthy adults is approximately 11.126 L/h, which is consistent with the 10.71 L/h reported in the literature (Song et al., 2020). The average renal clearance of imipenem in children with normal renal function is approximately 7.886 L/h. A single-dose clinical trial of imipenem in children with gram-negative bacillary infections showed that when 15 mg/kg of imipenem was infused, the clearance rate was 9.60 L/h in children aged 6–12 years and 5.31 L/h in children aged 2–6 years (Bradley et al., 2023). This is understandable because the physiological status of children changes with age and the renal clearance of imipenem varies with the administered dose. Nevertheless, individual simulation data show good consistency between our predicted data and the observed data, and the prediction errors of the model for PK parameters are all within a 1.5-fold range. In addition, we used the population simulation module to simulate the pharmacokinetic processes of virtual subjects for evaluating the model performance, and the dosing regimen used in the simulation was consistent with that in the relevant literature. The analysis results showed that all predicted values fell within the 90% confidence interval, and all observed data were within the range between the maximum and minimum predicted values. The GOF plot comparing predicted and observed values showed that all observations fell within the 2-fold error range of the predictions. These results indicated that the PBPK model had good stability and could accurately simulate the pharmacokinetic processes of imipenem in populations with normal renal function as well as in adult patients with RI. Therefore, the exposure of imipenem in pediatric patients with renal insufficiency can be accurately predicted using this model.

Similar to other carbapenem antibiotics, the antibacterial activity of imipenem depends on the percentage of time that the free drug concentration exceeds the minimum inhibitory concentration (%*f* T>MIC). It has been reported that to achieve antibacterial efficacy, the %*f* T>MIC generally needs to reach 50%-100% (Abdul-Aziz et al., 2020). In this study, the PK/PD target was set at 70% *f*T>MIC. In addition, according to the statistical data from the European Committee on Antimicrobial Susceptibility Testing (EUCAST), the epidemiological cutoff values of imipenem for most bacterial species, which reflect its antibacterial efficacy, range from 0.125 to 4 mg/L (Leclercq et al., 2013). Therefore, the focus of our study was to determine whether the pathogenic bacteria with the MIC ≤ 4 mg/L could meet the established target. The dosage of imipenem administered to children is typically based on body weight. According to the imipenem label, for pediatric patients older than 3 months, a dosage regimen of 15–25 mg/kg q6h is recommended (FDA, 2018). However, the label does not specify a recommended dosage for pediatric patients with RI. Based on PBPK model, the concentrations of imipenem after a 15 mg/kg infusion were predicted in this study for preschool children, school-age children, and adolescents with different renal function statuses. However, as shown in **Figure 6A**, the PTA for children with normal renal function receiving the dosage regimen of 15 mg/kg q6h (0.5 h infusion) was relatively low. Specifically, when the MIC was 1, 2, and 4 mg/L, the PTA values were only 57.86%, 48.81%, and 39.28%, respectively. Only when the MIC was 0.125 mg/L did the PTA exceed 80%. In children with mild RI, the attainment probability of the 15 mg/kg q6h (0.5 h infusion) regimen was relatively low, similar to that in children with normal renal function. For children with severe RI receiving the same regimen, the attainment probabilities are 100%, 99.52%, and 80% when MIC is 1, 2, and 4 mg/L, respectively. Notably, the therapeutic regimen of 15 mg/kg (0.5 h infusion) can only achieve therapeutic efficacy in children with severe RI, while it showed poor efficacy in children with normal renal function, mild RI, and moderate RI.

An international expert consensus points out that extended infusion of β-lactam antibiotics has significant advantages in reducing the emergence of drug resistance and improving the cure rate in critically ill patients. However, the consensus neither recommends nor opposes the extended infusion of β-lactam antibiotics for non-critically ill patients (Hong et al., 2023). The guidelines issued by the French Society of Pharmacology also recommend extending the infusion time of β-lactam antibiotics to achieve better therapeutic efficacy (Guilhaumou et al., 2019). A previous study conducted by our team also indicated that for renal-impaired patients undergoing continuous renal replacement therapy, extending the infusion time to 3 hours resulted in better antibacterial efficacy (Feng et al., 2025). Numerous other studies have also demonstrated that extending the infusion time can effectively increase the probability of β-lactam antibiotics reaching PD targets (De Waele et al., 2014; Langan et al., 2014; Tsai et al., 2016; Sjövall et al., 2018). Therefore, we adjusted the infusion time to 3 hours and reperformed dose extrapolation for pediatric patients receiving 15 mg/kg, with the resulting concentration-time profiles shown in **Figure 5B**. As shown in **Figure 6B**, for the dosage regimen of 15 mg/kg q6h with a 3-hour infusion, when the MIC is 2 mg/L, the probabilities of target attainment (PTAs) in children with normal renal function, mild RI, moderate RI, and severe RI were 90.24%, 92.62%, 99.52%, and 100%, respectively. When the MIC is 4 mg/L, the PTAs of the same regimen in children with normal renal function, mild RI, moderate RI, and severe RI were 78.57%, 80%, 86.19%, and 100%, respectively. These results indicated that extending the infusion time to 3 hours could enable children with different renal function statuses to achieve PK/PD target.

Subsequently, we further evaluated whether the adjusted dosage regimens for children with RI could achieve the same efficacy. As shown in **Table 3**, the predicted results indicated that the AUC values in children with mild RI, moderate RI, and severe RI were 1.05-fold, 1.26-fold, and 2.14-fold higher than those in children with normal renal function, respectively. Therefore, no dosage adjustment is required for children with mild RI, while the dosages for children with moderate RI and severe RI should be adjusted to 12 mg/kg q6h and 7 mg/kg q6h, respectively. **Figure 6C** presents the PTA profiles after dosage adjustment. When the 3-hour infusion regimen was administered at doses of 15 mg/kg q6h, 15 mg/kg q6h, 12 mg/kg q6h, and 7 mg/kg q6h for children with normal renal function, mild RI, moderate RI, and severe RI, respectively, the PTAs were 78.57%, 80%, 81.19%, and 85.71% at the MIC of 4 mg/L. Although the doses for children with moderate RI and severe RI were reduced, the corresponding antibacterial efficacy was still achieved, indicating that our dosage adjustments were acceptable.

This study also has several limitations. Currently, there is a lack of pharmacokinetic data on imipenem in pediatric patients with RI, making it impossible to validate the extrapolated concentration-time profiles of imipenem in these patients. Therefore, future studies need to accumulate relevant clinical data to further optimize this model. Disease states are complex processes. In this study, we only considered changes in drug elimination caused by renal insufficiency, while neglecting other physiological changes induced by RI. The study did not account for the impact of other concurrent medications on the PK processes. These unaccounted changes may not be reflected in the PBPK model. Thus, this study can only provide a rough dosage estimation for pediatric patients with RI. However, previous studies have established PBPK models for cyclosporine and ertapenem in children with renal insufficiency using similar methods (Yoon et al., 2019; Zhou et al., 2021). In addition, this study only uses “70%fT > MIC” as an indicator of clinical therapeutic efficacy. The assessment of clinical efficacy is a complex process that requires comprehensive evaluation based on other laboratory test indicators and pathogenic test results. Clinicians should flexibly adjust the dose based on the actual renal function, disease status, and therapeutic drug monitoring results of individual pediatric patients. Finally, this study is only applicable to children over 3 years old with normal growth and development, and not to neonates or children with RI who are overweight or underweight. Further research should be warranted to determine appropriate dosages for children with abnormal growth and development, as well as for infants under 3 years of age.

## Data Availability

The original contributions presented in the study are included in the article/**Supplementary Material**. Further inquiries can be directed to the corresponding author/s.
