# Supplementary material for: Physiologically based pharmacokinetic modeling and dose adjustment of imipenem in pediatric patients with renal impairment
Source: Front Cell Infect Microbiol. 2026 May 8;16:1798911. doi: 10.3389/fcimb.2026.1798911 (PMC13194417; doi:10.3389/fcimb.2026.1798911)
Supplement: Supplementary file 1 [file Table1.docx]

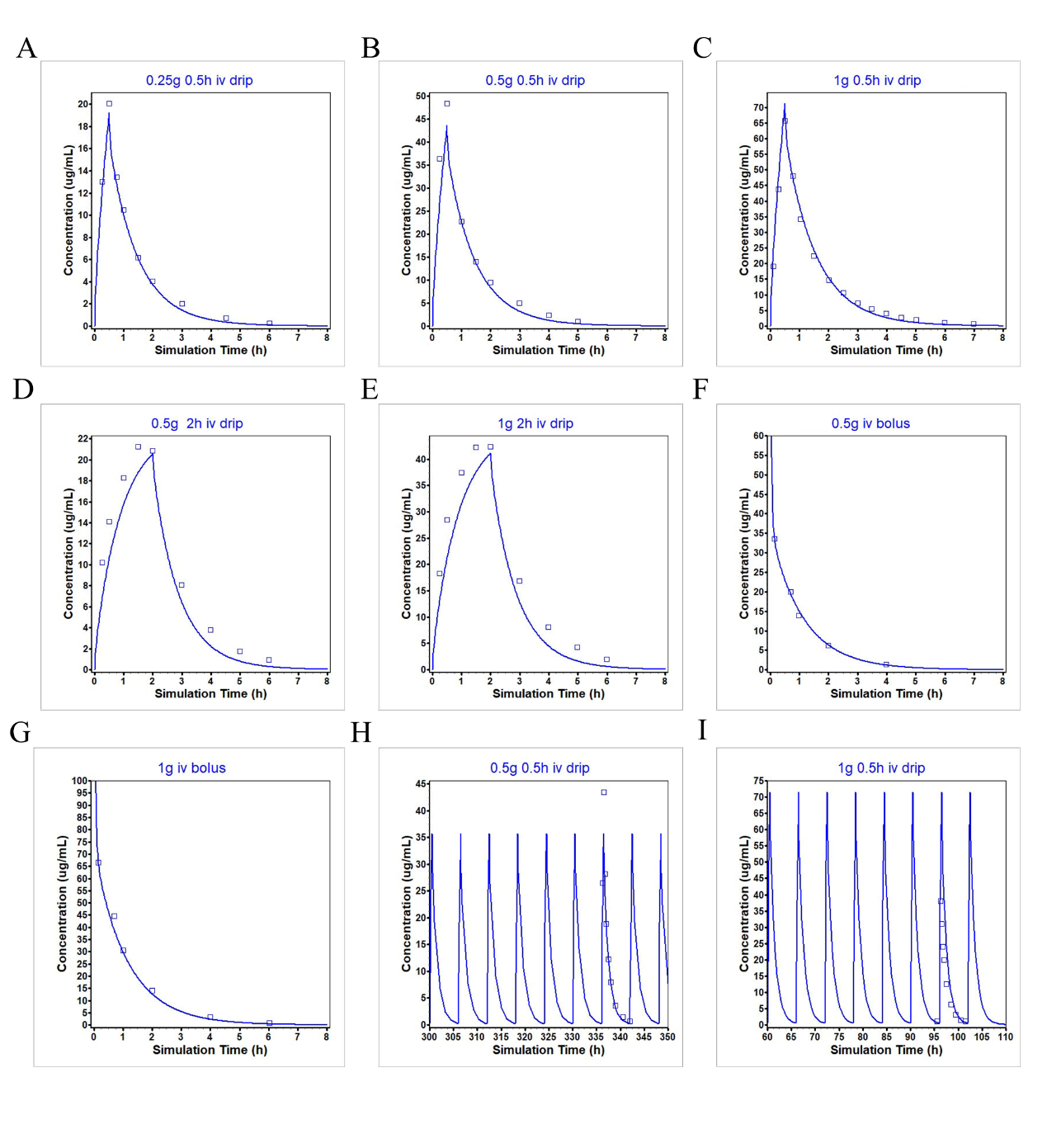


**Supplementary Figure S1.** The predictions of imipenem mean plasma concentration-time profiles after intravenous infusion in healthy adults (A-I), compared to their corresponding clinical data. Simulation results are shown as a blue line, observed values are shown as squares.


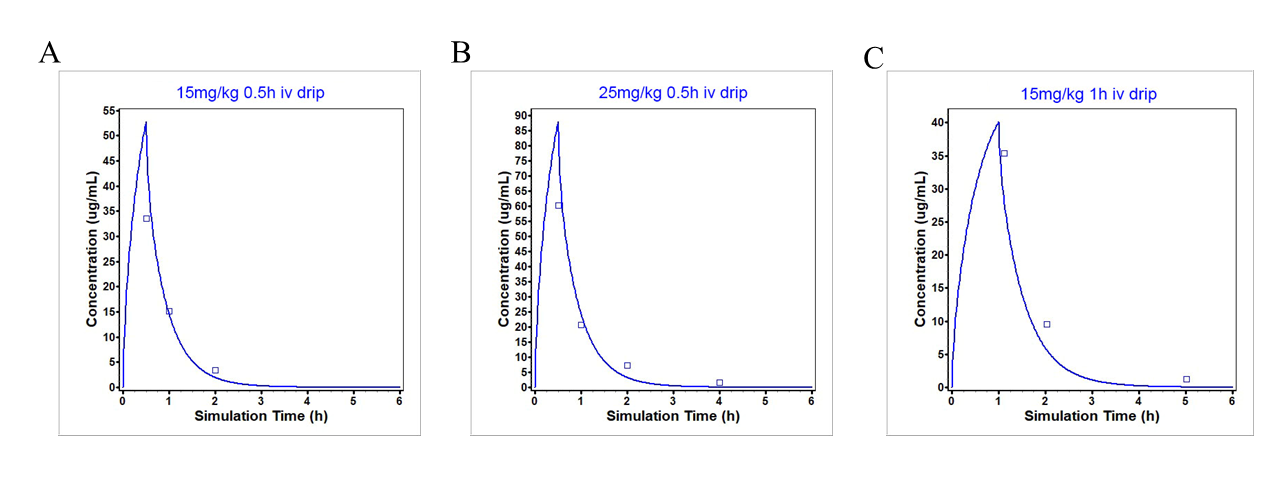


**Supplementary Figure S2.** The predictions of imipenem mean plasma concentration-time profiles after in children (A-C), compared to their corresponding clinical data. Simulation results are shown as a blue line, observed values are shown as squares.


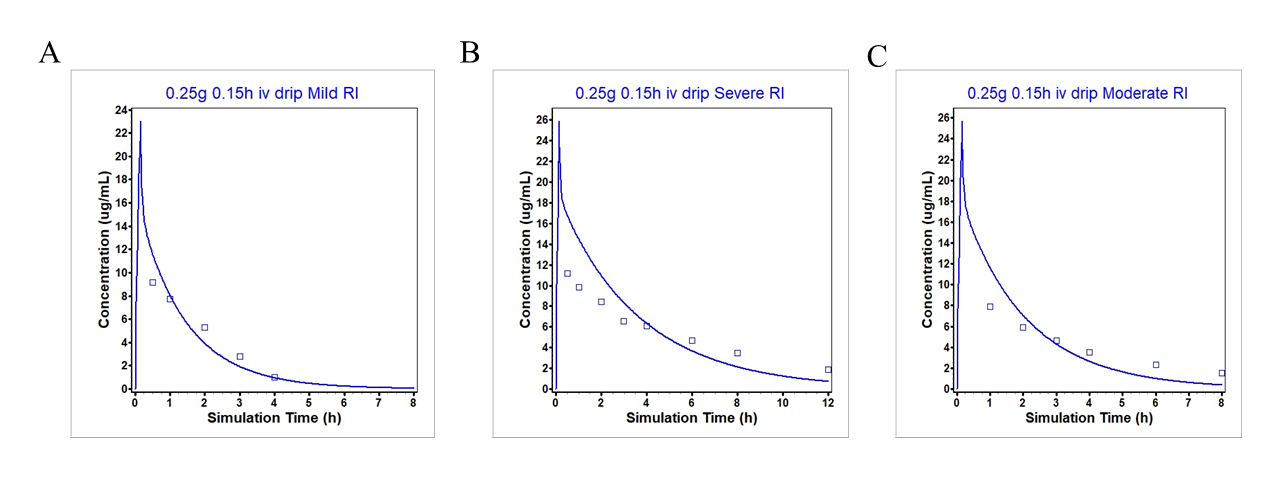


**Supplementary Figure S3.** The predictions of imipenem mean plasma concentration-time profiles after a single intravenous infusion in adults with mild (A), moderate (B), and severe (C) renal impairment, compared to their corresponding clinical data. Simulation results are shown as a blue line, observed values are shown as squares.


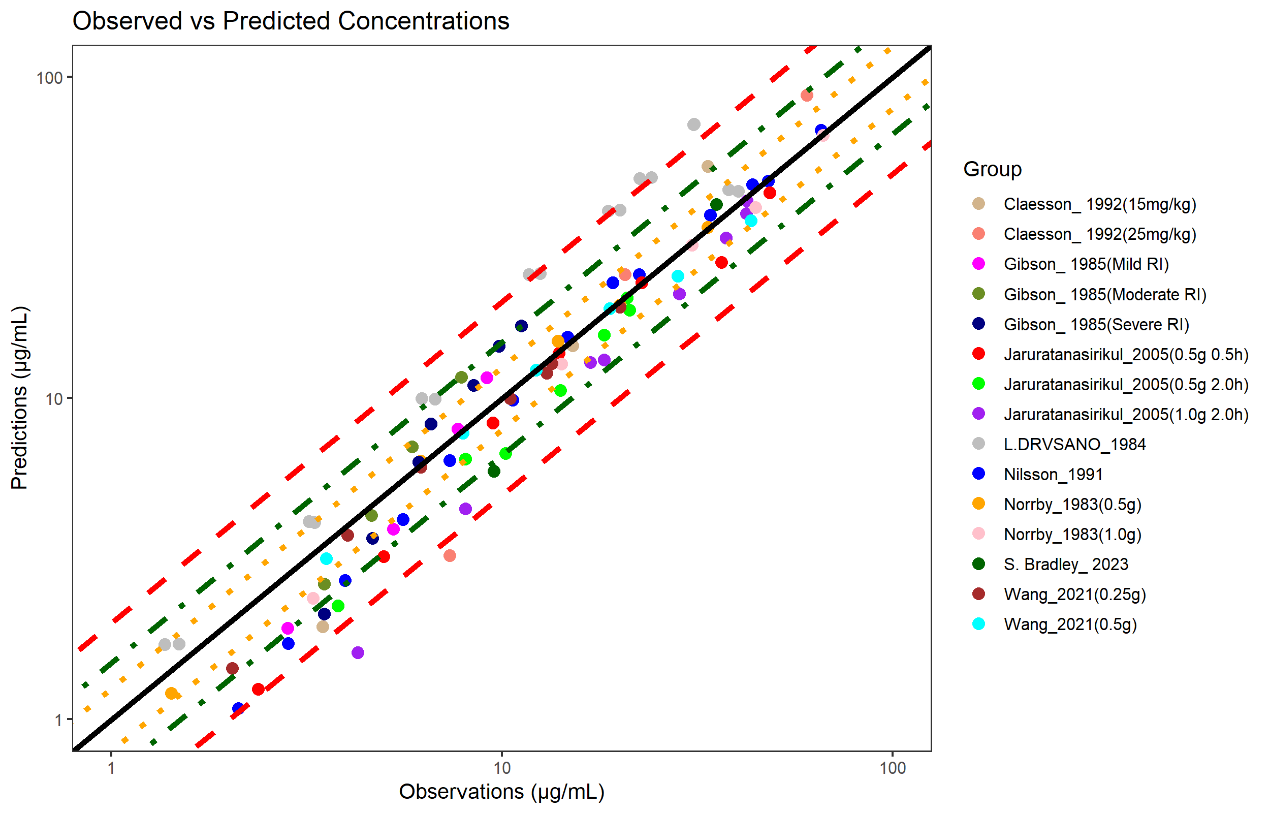


**Supplementary Figure S4.** Goodness-of-fit plot for plasma concentrations of imipenem in the populations used for PBPK modeling. The red dashed lines indicate the 2-fold error range, the dark green dashed lines indicate the 1.5-fold error range, and the orange dashed lines indicate the 1.25-fold error range. Colored circles represent the observed imipenem concentrations from different clinical trials.


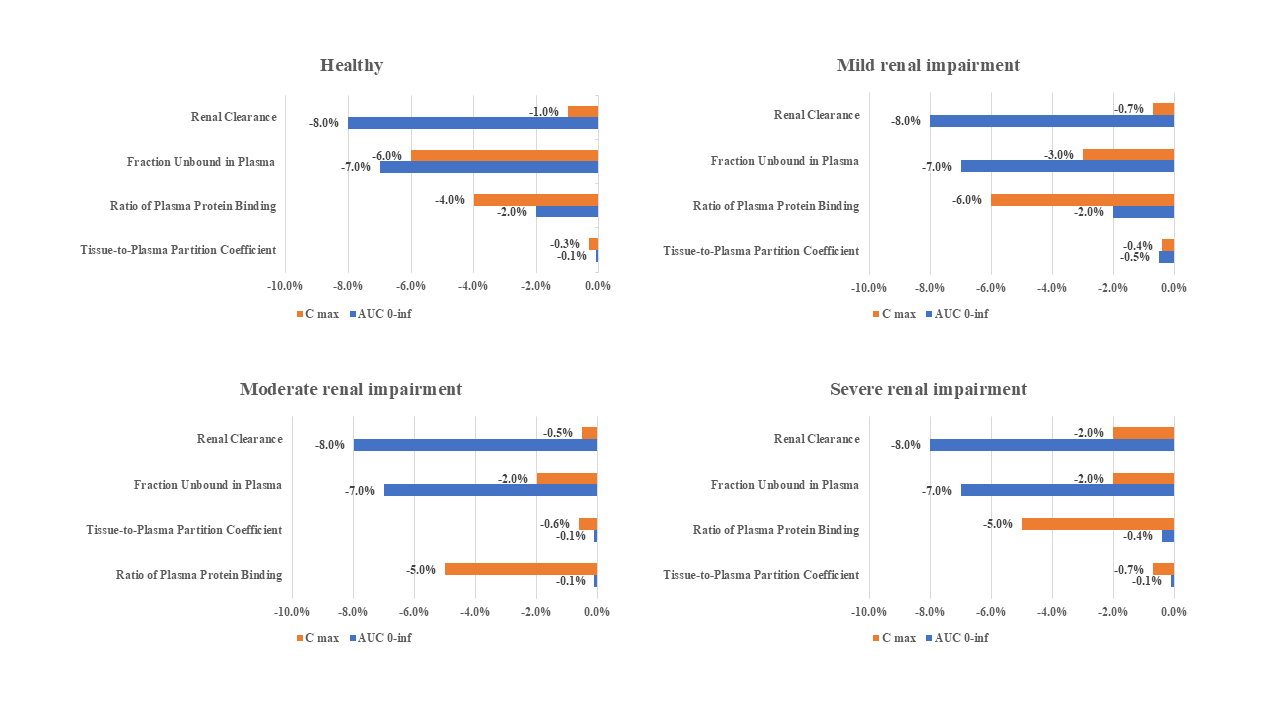


**Supplementary Figure S5.** Imipenem PBPK model sensitivity analysis in healthy population and renal impairment population. The sensitivity of each individual parameter was characterized by the changes in simulated AUC_0-inf_ and C_max_ following a 500 mg intravenous infusion over 30 minutes.

**Supplementary Table S1** The Clinical Pharmacokinetic Studies Used to Develop and Evaluate the Adult PBPK Model

| Study | Country | Renal function | Dose | Delivery path | Infusion time | N | Age, years  [mean/(rang)] | Weight, kg  [mean/(rang)] | Males (%) |
| --- | --- | --- | --- | --- | --- | --- | --- | --- | --- |
| Wang 2021[1] | China | Normal | 0.25 g | iv drip | 0.5 h | 12 | 34.2 (30-39) | 60.4 (52-69) | 41.7 |
| Jaruratanasirikul 2005[2] | Thailand | Normal | 0.5 g | iv drip | 0.5 h | 8 | 28.25 (24-39) | 58.75 (51-75) | 100 |
|  |  | Normal | 0.5 g | iv drip | 2.0 h | 8 | 28.25 (24-39) | 58.75 (51-75) | 100 |
|  |  | Normal | 1.0 g | iv drip | 2.0 h | 8 | 28.25 (24-39) | 58.75 (51-75) | 100 |
| Nilsson 1991[3] | Sweden | Normal | 1.0 g | iv drip | 0.5h | 8 | 33 (22-38) | 74 (66-86) | 100 |
| Norrby 1983[4] | Sweden | Normal | 0.5 g | iv | - | 8 | 25 (18-40) | 75 (60-89) | 100 |
|  |  | Normal | 1.0 g | iv | - | 8 | 25 (18-40) | 75 (60-89) | 100 |
| Wang 2021[1] | China | Normal | 0.5 g q6h^*^ | iv drip | 0.5 h | 12 | 34.2 (30-39) | 60.4 (52-69) | 41.7 |
| Drusano 1984[5] | America | Normal | 1.0 g q6h^*^ | iv drip | 0.5 h | 6 | - (19-34) | - (54.5-85.5) | 100 |
| Gibson 1985[6] | America | Mild RI | 0.25 g | iv drip | 0.15 h | 6 | 60.8 (44-68) | 86.6 (67.3-116.0) | 100 |
|  |  | Moderate RI | 0.25 g | iv drip | 0.15 h | 6 | 47.7 (25-60) | 76.5 (64.5-92.0) | 100 |
|  |  | Severe RI | 0.25 g | iv drip | 0.15 h | 6 | 53.1 (45-58) | 71.3 (60.0-82.5) | 100 |

RI, renal impairment; *, Multi-dose; N, number of volunteers

**Supplementary Table S2** The Clinical Pharmacokinetic Studies Used to Develop and Evaluate the Pediatric PBPK Model

| Study | Country | Renal function | Dose | Delivery path | Infusion time | N | Age, years [mean/(rang)] | Weight, kg [mean/(rang)] | Males (%) |
| --- | --- | --- | --- | --- | --- | --- | --- | --- | --- |
| Claesson 1992[7] | Sweden | Normal | 15 mg/kg | iv drip | 0.5 h | 8 | -（3-12） | - | - |
|  |  | Normal | 25 mg/kg | iv drip | 0.5 h | 2 | -（3-12） | - | - |
| S. Bradley 2023[8] | America | Normal | 15 mg/kg | iv drip | 1.0 h | 6 | -（2-6） | 15.4 (12-19) | 33.3% |

N, number of volunteers

**Supplementary Table S3** Physiological and Drug Factors of Population.

| Study | Renal function | Dose | CL(L/h) | Vss(L/kg) | T1/2(h) | GFR (mL/s) | QR(mL/s) | QUR | Fup |
| --- | --- | --- | --- | --- | --- | --- | --- | --- | --- |
| Wang 2021[1] | Normal | 0.25 g | 10.972 | 10.447 | 0.660 | 1.6267 | 19.6 | 0.0228 | 0.8 |
| Jaruratanasirikul 2005[2] | Normal | 0.5 g | 10.726 | 9.135 | 0.590 | 1.5996 | 17.763 | 0.0197 | 0.8 |
|  | Normal | 0.5 g | 10.726 | 9.135 | 0.590 | 1.5996 | 17.763 | 0.0197 | 0.8 |
|  | Normal | 1.0 g | 10.726 | 9.135 | 0.590 | 1.5996 | 17.763 | 0.0197 | 0.8 |
| Nilsson 1991[3] | Normal | 1.0 g | 11.126 | 11.663 | 0.723 | 1.9632 | 21.35 | 0.0233 | 0.8 |
| Norrby 1983[4] | Normal | 0.5 g | 11.262 | 12.371 | 0.761 | 2.0051 | 22.168 | 0.0252 | 0.8 |
|  | Normal | 1.0 g | 11.262 | 12.371 | 0.761 | 2.0051 | 22.168 | 0.0252 | 0.8 |
| Wang 2021[1] | Normal | 0.5 g q6h^*^ | 11.176 | 11.663 | 0.723 | 1.9632 | 21.356 | 0.0233 | 0.8 |
| Drusano 1984[5] | Normal | 1.0 g q6h^*^ | 11.176 | 11.663 | 0.723 | 1.9632 | 21.356 | 0.0233 | 0.8 |
| Gibson 1985[6] | Mild RI | 0.25 g | 10.682 | 14.081 | 0.913 | 1.3944 | 19.188 | 0.0295 | 0.80588 |
|  | Moderate RI | 0.25 g | 6.376 | 12.275 | 1.334 | 0.4449 | 20.692 | 0.0243 | 0.82723 |
|  | Severe RI | 0.25 g | 3.486 | 12.194 | 2.424 | 0.1433 | 19.216 | 0.024 | 0.83352 |
| Claesson 1992[7] | Normal | 15 mg/kg | 7.886 | 3.259 | 0.286 | 1.0213 | 6.7561 | 0.0067 | 0.81217 |
|  | Normal | 25 mg/kg | 7.886 | 3.259 | 0.286 | 1.0213 | 6.7561 | 0.0067 | 0.81217 |
| S. Bradley 2023[8] | Normal | 15 mg/kg | 8.489 | 4.221 | 0.345 | 1.1379 | 8.0532 | 0.0067 | 0.81182 |

RI, renal impairment; *, Multi-dose; N, number of volunteers; CL, total clearance; V_ss_ , volume of distribution at steady-state; GFR, glomerular filtration rate; QR, renal blood flow; QCR, urine flow rate; *^a^*, normal renal function; RI, renal impairment

**References**

[1]. Wang X, Liu N, Wei Y, Zhang S, Li H, Yan B, et al. A Single- and Multiple-Dose Study To Characterize the Pharmacokinetics, Safety, and Tolerability of Imipenem and Relebactam in Healthy Chinese Participants. Antimicrobial agents and chemotherapy. 2021;65(3). DOI: 10.1128/aac.01391-20.

[2]. Jaruratanasirikul S, Raungsri N, Punyo J, Sriwiriyajan S. Pharmacokinetics of imipenem in healthy volunteers following administration by 2 h or 0.5 h infusion. The Journal of antimicrobial chemotherapy. 2005;56(6):1163-5. DOI: 10.1093/jac/dki375.

[3]. Nilsson-Ehle I, Hutchison M, Haworth SJ, Norrby SR. Pharmacokinetics of meropenem compared to imipenem-cilastatin in young, healthy males. European journal of clinical microbiology & infectious diseases : official publication of the European Society of Clinical Microbiology. 1991;10(2):85-8. DOI: 10.1007/bf01964413.

[4]. Norrby SR, Björnegård B, Ferber F, Jones KH. Pharmacokinetics of imipenem in healthy volunteers. The Journal of antimicrobial chemotherapy. 1983;12 Suppl D:109-24. DOI: 10.1093/jac/12.suppl_d.109.

[5]. Drusano GL, Standiford HC, Bustamante C, Forrest A, Rivera G, Leslie J, et al. Multiple-dose pharmacokinetics of imipenem-cilastatin. Antimicrobial agents and chemotherapy. 1984;26(5):715-21. DOI: 10.1128/aac.26.5.715.

[6]. Gibson TP, Demetriades JL, Bland JA. Imipenem/cilastatin: pharmacokinetic profile in renal insufficiency. The American journal of medicine. 1985;78(6a):54-61. DOI: 10.1016/0002-9343(85)90102-0.

[7]. Claesson G, Eriksson M, Rogers JD. Pharmacokinetics of imipenem/cilastatin sodium in children with peritonitis. Pharmacology & toxicology. 1992;71(2):103-6. DOI: 10.1111/j.1600-0773.1992.tb00527.x.

[8]. Bradley JS, Makieieva N, Tøndel C, Roilides E, Kelly MS, Patel M, et al. Pharmacokinetics, Safety, and Tolerability of Imipenem/Cilastatin/Relebactam in Children with Confirmed or Suspected Gram-Negative Bacterial Infections: A Phase 1b, Open-Label, Single-Dose Clinical Trial. Journal of clinical pharmacology. 2023;63(12):1387-97. DOI: 10.1002/jcph.2334.
